# Effect of web-based training on public health nurses’ program implementation capacity: a randomized controlled trial

**DOI:** 10.1186/s12912-024-02287-z

**Published:** 2024-09-27

**Authors:** Keiko Miyamoto, Reiko Okamoto, Keiko Koide, Mirei Shimodawa

**Affiliations:** 1 Osaka University Graduate School of Medicine, Division of Health Sciences, Yamadaoka 1-7, Suita-city, Osaka, 565-0871 Japan; 2 Former Osaka University Graduate School of Medicine, Division of Health Sciences, Suita, Japan

**Keywords:** Dissemination and implementation science, Capacity building, Public health nurses, Simulation, Web-based training

## Abstract

**Background:**

Health service development aims to close the gap between evidence and practice by adopting and appropriately utilizing the latest findings. To address this gap, dissemination and implementation research has been promoted and developed. Despite promoting evidence-based programs over the years, public health nurses have had few training opportunities in evidence-based public health. This study evaluated the effectiveness of a web-based training in building the basic program implementation capacity of public health nurses with two to five years of experience.

**Methods:**

We developed a simulation-powered web-based training according to an Implementation Degree Assessment Sheet for health programs. This was a randomized, single-blind, parallel-group trial. The primary outcome, the result of implementation capacity for public health nurses, was assessed by the total score of the Implementation Degree Assessment Sheet, and scores for its five domains. The secondary outcome was evaluated by the level of understanding. The primary outcome was analyzed utilizing the t-test and analysis of covariance, whereas the secondary outcome was assessed utilizing the U-test and Quade’s analysis of covariance. Data were collected directly before intervention (T1: baseline), immediately post-intervention (T2), and four weeks post-intervention (T3: endpoint).

**Results:**

The 197 participants were randomly allocated to either the intervention group (*n =* 98) or the control group (*n =* 99). A full analysis set of 152 samples and a per-protocol set of 104 samples were analyzed. The intervention group exhibited a significantly higher total score and five domain-wise scores at the endpoint compared with the control group. The disparity between the endpoint and baseline scores was significantly larger for the intervention group for all scores. The level of understanding was significantly higher in the intervention group than in the control group at T2 and T3. The effect size of the total score was higher in the full analysis set (Cohen’s d = 0.5) than in the per-protocol set (d = 0.48).

**Conclusions:**

This web-based training was effective in building the program implementation capacity of participants four weeks post-intervention.

**Trial registration:**

University Hospital Medical Information Network Center Clinical Trials Registry UMIN000048421.

**Supplementary Information:**

The online version contains supplementary material available at 10.1186/s12912-024-02287-z.

## Background

Health-service development involves bridging the gap between evidence and practice by adopting and appropriately utilizing the latest findings [1]. To address this gap, dissemination and implementation (D&I) research has been actively promoted and developed [2]. D&I is defined as follows: “Dissemination research is the systematic study of processes and factors that lead to widespread use of an evidence-based intervention by the target population” [3] and “Implementation research is the scientific study of methods to promote the integration of research findings and evidence-based interventions into healthcare policy and practice” [4]. However, a survey conducted in Europe and America found that only 50–60% of participants in evidence-based public health (EBPH) training had implemented any EBPH program in their workplace [5], which is unsatisfactory. This situation in countries where EBPH has been promoted indicates that both practitioners and academics still have a long way to go in closing the gap between evidence and practice.

In Japan, government-led efforts to promote evidence-based programs have been inadequate in supporting frontline staff. The Cabinet Office launched the Economic and Fiscal Revitalization Action Program in 2015 to encourage program development and build evidence-based best practices [6]. However, little progress has been made toward achieving the desired objective of developing human resources to drive the D&I of best practices on the frontline, particularly for public health nurses (PHNs).

PHNs, who play a key role in promoting public health in Japan, face multiple challenges, partly because of ongoing generational transitions. According to the national guidance on PHN activities, PHNs working for local governments are crucial in maintaining and enhancing the health of individuals and communities [7]. As of 2022, there were approximately 37,000 PHNs, with almost 36% having only one to six years of experience [8]. Although over 90% of PHNs have received undergraduate education since 2015 [9], they face numerous challenges when dealing with complex and diverse health issues. Further, they lack confidence, primarily because of the lack of opportunities to learn skills in EBPH program development [10]. In a field survey [11], 70% of the PHNs requested support for implementing evidence-based programs, regardless of their related experience. These findings indicate that PHNs require initiatives to build their capacities for implementing evidence-based programs.

However, various barriers to building program implementation capacity exist at the individual, institutional, and governmental levels. Common difficulties include insufficient funding, incentives, and time; challenges for practitioners in terms of finding, choosing, and applying evidence; and inadequate support at the workplace, institutional, and government levels [12–15]. The situation is similar in Japan, highlighting the need to develop programs that enable capacity building at the individual level.

Simulation-powered training may be an effective means of capacity building for public health practitioners. As a type of active learning, simulation is considered essential for competency-based education, offering in-depth learning opportunities by enabling learners to think independently, deepen their understanding, and apply the knowledge gained [16]. Simulation programs are effective in enhancing public health students’ capabilities, including evaluation, critical thinking, communication, and collaboration with service recipients and their families [17]. These capabilities are also crucial for program-implementation training.

The D&I research framework can guide practitioners in implementing the EBPH. The Consolidated Framework for Implementation Research (CFIR), a systematic guiding tool for building program implementation capacity, is reported to be capable of evaluating whether current or planned programs will be adequate or successful by providing an adaptable framework for the implementation context [18]. Building on the CFIR, Okamoto et al. [19] developed an assessment sheet for program development specifically for PHNs. This sheet, called the Implementation Degree Assessment Sheet (IDAS), is considered a valuable teaching aid as it not only serves as a practical outcome measure for program implementation but also as a measure for evaluating the implementation capacity of PHNs themselves [19].

There is an urgent need to develop and provide online implementation programs for new PHNs based on the D&I theory. As mentioned earlier, PHNs in Japan have minimal opportunities for training in program implementation, despite their key role in public health activities. Although Yoshioka-Maeda et al. [20] developed a web-based program for mid-career PHNs to implement health program planning, there is no training program specifically for new PHNs to practice D&I-based EBPH. Implementation programs based on D&I are essential for new or inexperienced PHNs, who constitute almost 40% of the PHNs working for municipalities, to address complex health issues effectively. These programs must allow busy professionals to acquire skills and knowledge at their own pace.

This study evaluated the effectiveness of a web-based training program in building the basic program implementation capacity of PHNs with two to five years of experience.

## Methods

### Design and randomization

This was a randomized, single-blind, parallel-group trial. Years of experience as a PHN (two to three years, four to five years) and affiliation (prefectures, government-designated or other major cities, other municipalities) were utilized for allocation purposes employing a stratified permuted block method. The allocation table developed by the author was uploaded to the Research Electronic Data Capture (RED-Cap) system and immediately deleted to prevent any subsequent arbitrary allocation. Participants were automatically assigned by the RED-Cap to the intervention or control group [1:1 ratio] based on the order of registration. The intervention group viewed our program, whereas the control group did not receive any intervention in the trial. Owing to ethical considerations, participants allocated to the control group were given an opportunity to receive the same training after completing the questionnaires.

The research manager then sent the URL, password, and training instructions for the program to the participants’ personal e-mail addresses. Participants were strictly prohibited from disclosing the URL, password, or other information about the program to third parties. The study was reported in accordance with the Consolidated Standards of Reporting Trials (CONSORT) [21] and the Template for Intervention Description and Replication (TIDieR) guidelines [22].

### Eligibility criteria

Participants were selected from PHNs working full-time at public health offices with two to five years of experience who were not on sick, maternal, or parental leave. Eligible participants needed to be available to complete the program and have access to the program from their own devices, including smartphones and personal computers.

### Sample and setting

Based on the findings of Yoshioka–Maeda et al. [20], a sample size of 134 was calculated utilizing G*power 3.1.9.2, where the effect size, alpha, and power were set at 0.5, 0.05%, and 80%, respectively. However, based on the findings of their research, it was determined that 174 samples were required to counter a dropout rate of 30%.

To solicit participation, survey documents were sent by mail to 53 prefectural health centers, 123 health centers controlled by government-designated cities or major cities, and 138 municipal health centers in the Kansai, Chubu, and Chugoku regions of Japan in mid-October 2022. Additionally, booklets outlining this study were sent so that PHNs could apply for participation based on fully comprehending the study. Applicants were asked to register on the RED-Cap webpage by early December 2022.

### Intervention

This program was named the Capacity Development Training Course for Evidence-based Program Implementation (“EPI-TRE”). The ultimate objective of this program was to acquire basic program implementation capacity. The IDAS was adopted as a training framework because, as a competency list, it enables the assessment of individual program implementation capacities [19].

The IDAS was developed utilizing the three phases proposed by Boateng et al. [23]. Initially, a validated implementation science framework suitable for this program was selected [19]. The CFIR, selected as the framework, was translated into Japanese and verified multiple times for accuracy [19]. To ensure that the framework’s content was appropriate for the context of Japanese health programs, multiple revisions were made through expert consultation and pre-testing [19]. In the second phase, a questionnaire survey was administered to the target population nationwide as the primary test [19]. In the final phase, the reliability and validity of this scale were examined from the obtained data (GFI: 0.87, CFI: 0.92, RMSEA: 0.06, Cronbach’s alpha > 0.8, Spearman’s coefficient 0.95), all reaching acceptable levels [19].

The IDAS lists 31 core indicators across five domains to assist PHNs with implementing evidence-based programs [19]. The five domains are defined as: 1) “Intervention characteristics,” referring to activities to verify or adjust the evidence, merits, procedures, adaptability to the local context, expenses, and so on of a program; 2) “Outer setting” referring to activities to identify and leverage external factors, including extrinsic incentives, best practice, and possibilities of collaboration; 3) “Inner setting,” referring to activities to encourage program introduction by setting or verifying objectives, considering organizational culture and the impact of other intrinsic factors; 4) “Characteristics of individuals,” referring to the competencies of individuals, measured regarding self-efficacy, professional identity, and ongoing upskilling; and 5) “Process,” involving activities to plan, implement, review, and evaluate a program at both the individual and organizational levels, in collaboration with other stakeholders [24].

Three D&I researchers with public health nursing experience (two of whom created the IDAS) discussed a framework for the training program based on the 4-step implementation science framework [25], the 10-step learning derivation of policy transfer [26], and IDAS. Consequently, the framework for this training was set at eight items. Additionally, the research group decided to focus on three indicators within the five domains that PHNs with five years or less experience had been found to place less importance on, and therefore have fewer opportunities to implement, based on a prior survey by Okamoto et al. [27]. These domains include “verification of evidence,” “verification of trialability,” and “access to knowledge and information” [27]. The training was drafted by the research group over approximately one month, with input from five health program experts who also participated in the creation of the IDAS. Currently, EPI-TRE is accessible at https://www.phn-waza.com/content2-3/ [28].

The program consists of four modules: the introduction (Module 0) and three competency-building modules (Modules 1–3). Each training module lasts approximately 30 minutes and is divided into several parts, enabling participants to start from any part. All parts remain accessible for four weeks, during which participants can view the content as many times as they like. However, as the program does not allow fast-forwarding, it requires the complete viewing of each part. The program features animated characters as lecturers, facilitators, and supporters to guide the participants through the learning process. It includes narrative simulations where participants are asked to select their preferred conduct from two options in each situation.

An outline of the web-based training program is presented in Table 1. Module 1 aims to incentivize participants to engage in the program by assisting them with comprehending the basic facts and skills regarding program implementation. This module includes simulations of the eight steps of program implementation and a lecture on evidence. It also builds on Lave’s learning transfer model [29].

Module 2 was designed to enhance the participants’ readiness to implement EBPH programs. In this module, participants learn how to introduce best practices by leveraging the most appropriate knowledge and skills. It includes simulations for capacity-building related to the “quality and level of evidence” and “verification of trialability.” The lecture in Module 2 focuses on “access to knowledge and information.”

Module 3 has the same objective as Module 2, enabling participants to review what they have learned. If participants answer a question incorrectly, they are automatically directed back to the relevant page for re-learning. A message encouraging participants is included at the end of the module. Modules 2 and 3 are based on an experimental learning model [30]. As part of the engagement strategy of the study, reminder e-mails were sent to all participants to advise them when to complete the survey. Thereafter e-mails were frequently sent to non-participants to encourage their participation and ensure all participants completed the program, including the questionnaires. The e-mail messages were changed several times to increase their motivation. The program also used animated characters and music to encourage participation. As a further incentive, a downloadable PDF file for the program was sent to those who completed all the activities. In line with the findings of Yoshida-Maeda et al. [20], we did not organize any group sessions but provided support for access to the program.

**Table 1:** Components of the training program

Module	Minutes	Title	Contents	Simulations of EBPH program implementation
0	4min	Introduction	1) Explain in the way to use the training program	
			2) Introduce terms and definitions that are used in the training	
1	28min	Understand the main point of health program implementation	1) Clarify the necessity of developing or improving an EBPH program	Family support program
			2) Share the necessity of developing or improving a program among stakeholders and introduce a project	
			3) Select an appropriate best practice case	
			4) Establish evidence supporting the best practice program selected	
			5) Decide on whether to adopt a program and prepare for its application	
			6) Increase the possibility of successful implementation of the adopted program	
			7) Evaluate the new or improved program after implementation	
			8) Build professional competencies	
			Lecture 1: What is evidence? / How to use evidence	
2	27min	Apply the best practice to new program	1) Review module 1	Community health promotion program
			2) How to select an appropriate best practice case	
			3) How to establish evidence supporting the best practice program selected	
			4) How to decide to adopt a best practice/ How to prepare for its application	
			Lecture 2: How to find evidence	
3	35min	Use the program in your own practice	1) Review module 2/How to study module3	Disaster management for community
			Lecture 3: Positive outcome resulting from the development of the implementation skill	
			2) Clarify the necessity of developing or improving an EBPH program	
			3) Share the necessity of developing or improving a program among stakeholders, and introduce a project	
			4) Select an appropriate best practice case	
			5) Establish evidence supporting the best practice program selected	
			6) Decide on whether to adopt a program and prepare for its application	
			7) Increase the possibility of successful implementation of the adopted program	
			8) Evaluate the new or improved program after implementation	
			9) Encourage the participants to build professional competencies	

### Outcome measures

#### Primary outcome

The IDAS scale measures the competencies of PHNs to implement an EBPH program with proven reliability and validity [19]. Respondents answer questions about their competencies (knowledge, skills, and attitudes) on a scale of 0 (never) to 5 (always) [19]. Data on the total IDAS score and scores for the five domains of the IDAS scale were collected at three points: Time 1 (directly prior to intervention: baseline), Time 2 (immediately post-intervention), and Time 3 (four weeks post-intervention: endpoint).

#### Secondary outcome

Questions were developed to assess the extent to which the participants understood the program based on the eight items of the program framework. These questions aimed to identify the level of knowledge regarding 1) how to clarify the necessity of developing or improving an EBPH program; 2) how to share the necessity of developing or improving a program among stakeholders, and introducing a program 3) how to select an appropriate best practice case; 4) how to establish evidence supporting the selected best practice program; 5) how to decide whether to adopt a program and prepare for its application; 6) how to increase the possibility of successful implementation of the adopted program; 7) how to evaluate the new or improved program post implementation; and 8) how to build professional competencies. Participants rated their understanding on a scale of 0 (insufficient) to 10 (sufficient). These responses were collected simultaneously with the IDAS scores. The internal consistency of this measure, as assessed by Cronbach’s alpha, was 0.988.

### Process evaluation

The duration of program viewing was monitored to track the participants’ progress. Satisfaction with and confidence in the program were measured using the Japanese version of the Simulation Design Scale [31], with a modified response scale: 1 (strongly disagree or disagree), 2 (do not know), and 3 (agree or strongly agree). Five questions assessed satisfaction, and eight assessed confidence, with responses collected at T2. Participants were also asked to provide their impressions of the training and any additional comments upon completion of each of the three competence-building modules.

### Baseline characteristics

We gathered demographic information about the participants at T1. Specifically, data were collected regarding sex, age, years of experience as a PHN, affiliation, experience in implementing EBPH programs, and academic qualifications.

### Statistical analysis

To compare participant demographics between the control and intervention groups, Fisher’s exact test was utilized for categorical variables and the Mann–Whitney U-test for continuous variables. Following the intent-to-treat principle, analyses were conducted utilizing both a full analysis set (FAS) and a per-protocol set (PPS). The FAS included participants who completed the baseline questionnaire and proceeded to Module 1 of the intervention program. Missing data were not imputed, as indicated by Little’s missing completely at random test (χ^2^ = 19.96, DF = 20, *P =* 0.460). The primary outcome, the total IDAS score, was analyzed utilizing t-tests at T1, T2, and T3. Similarly, scores for the five domains of the IDAS were analyzed. Adjustments for differences between T1 and T3 scores in years of experience and affiliation were conducted utilizing analysis of covariance (ANCOVA). The level of understanding was assessed at each time point utilizing the Mann–Whitney U-test. Differences in scores between the baseline and endpoint were adjusted for years of experience and affiliation utilizing Quade’s non-parametric ANCOVA test. Statistical analysis was conducted utilizing IBM SPSS Statistics for Windows ver. 29. All statistical tests were two-tailed, and values less than 0.05 were considered statistically significant.

### Ethical considerations

This study was approved by the Ethics Committee for the Intervention Study of Osaka University Hospital (Nos. 22087 and 21/7/2022), and all procedures were conducted in accordance with the Declaration of Helsinki. Participants who ticked the “I agree” box on the web-based application were deemed to have provided consent to participate in this study. Regarding the minimum data required for the study, information on the type and name of the affiliated institution, years of experience as a PHN, and any considerations for inclusiveness were collected. The name of the affiliated institution was obtained to create an accurate allocation table for stratified permuted block randomization and minimize the amount of personal information to be collected. The information obtained was anonymized utilizing IDs and stored separately in lockable shelves. This study was registered in the University Hospital Medical Information Network Center Clinical Trials Registry (UMIN000048421:18/06/2022).

## Results

### Study participants

**Fig. 1:**
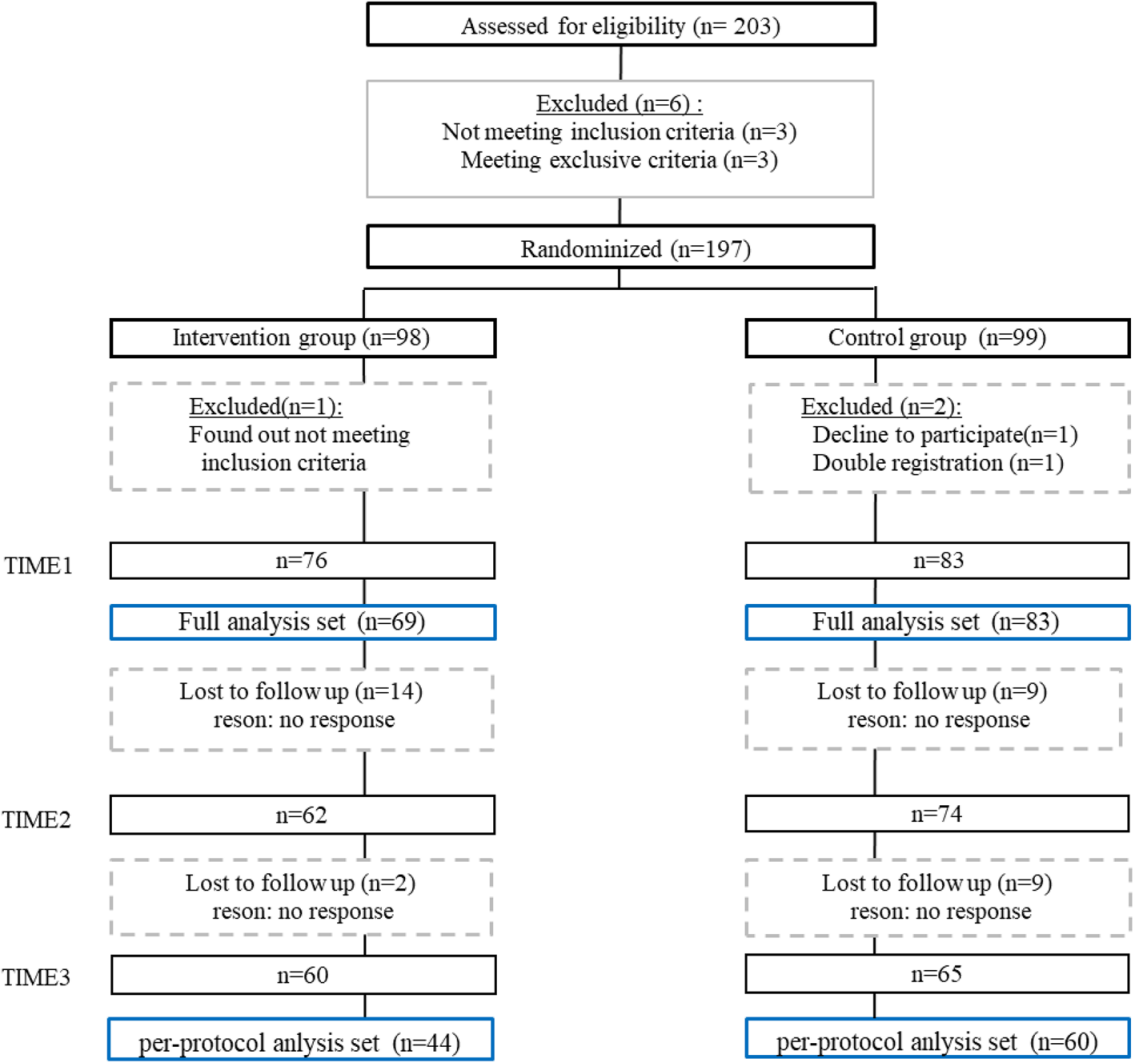
Flow of study participants

Figure 1 illustrates the program enrollment details following CONSORT guidelines [21]. As a result of the onset of the eighth wave of the COVID-19 pandemic in Japan just before the program’s commencement, enrolment was continued after reaching the target number in anticipation of potential dropouts. A total of 203 PHNs initially registered for participation, with six found ineligible: three, because of lack of consent; two, for not meeting the full-time criteria; and one, with more than six years of experience. Among the remaining 197 participants, 98 were allocated to the intervention group and 99 to the control group. One participant in the intervention group was later found ineligible, while two participants in the control group withdrew—one because of technical issues and another for registering with multiple e-mail addresses. Consequently, the program began with 97 participants in both groups. However, by the fourth week, 37 participants in the intervention group and 32 in the control group dropped out for unspecified reasons. The study was conducted from October 20, 2022, to March 20, 2023.

According to the protocol, the PPS included 44 participants in the intervention group and 60 in the control group. The FAS, comprising participants who completed the questionnaire at T1 and proceeded to Module 1 for skill-building, included 69 participants in the intervention group and 93 in the control group.

### Baseline characteristics of study participants

Table 2 exhibits the participants’ demographics. The mean age of participants was 29.42 in the intervention group and 28.58 in the control group. Inexperienced PHNs in EBPH programs accounted for 76.3% in the intervention group and 59% in the control group, indicating a significantly higher proportion of inexperienced PHNs in the intervention group. No other significant disparities were observed between the two groups.

**Table 2:** Demographic characteristics of the intervention and control groups at baseline

Characteristics	Intervention group(*n*=76)		Control group(*n*=83)		
	Mean/n		Mean/n		p
Age (SD)	29.42	(6.3)	28.58	(5.1)	0.702
Sex (%)					
Female	69	(90.8)	80	(96.3)	0.196
Male	7	(9.2)	3	(3.6)	
Years of experience as a public health nurse (%)					
2years	24	(31.6)	33	(39.8)	0.551
3years	22	(28.9)	19	(22.9)	
4years	16	(21.1)	20	(24.1)	
5years	14	(18.4)	11	(13.3)	
Affiliation (%)					
Prefectures	24	(31.6)	30	(36.1)	0.814
Government-designated cities or major cities	28	(36.8)	27	(32.5)	
Other municipalities	24	(31.6)	26	(31.3)	
Experience in EBPH project implementation (%)					
No	58	(76.3)	49	(59.0)	0.028
Yes	18	(23.7)	34	(41.0)	
Academic qualifications (%)					
Vocational training school	1	(1.3)	2	(2.4)	0.833
Junior college	5	(6.6)	7	(8.4)	
Bachelor’s Degree	66	(86.8)	69	(83.1)	
Master’s Degree	3	(3.9)	5	(6.0)	
Doctorate Degree	1	(1.3)	0		

*P*-values: Mann-Whitney U test for continuous variables, Fisher’s exact test for categorical variables

### Main outcome

Table 3 exhibits the results of the main outcome. The t-test results indicated that the mean ±SD of the total IDAS scores at the endpoint was significantly higher in the intervention group (97.98 ± 27.15) compared to the control group (85.05 ± 24.48) [t(123) = 2.801, *p =* 0.006]. Similarly, significant differences were observed between the intervention and control groups across all five domains of the IDAS at the endpoint (*p <* 0.030). Specifically, the mean ± SD scores for the intervention group compared to the control group were as follows: intervention characteristics (25.07 ± 7.07 vs. 22.37 ± 6.67, t(123) = 2.194), outer setting (12.25 ± 3.98 vs. 10.74 ± 3.48, t(123) = 2.265), inner setting (28.60 ± 8.07 vs. 24.29 ± 7.54, t(123) = 3.085), characteristics of individuals (12.62 ± 3.92 vs. 10.72 ± 3.76, t(123) = 2.753), and process (19.45 ± 6.06 vs. 16.92 ± 5.09, t(123) = 2.530).

**Table 3:** The study outcomes for both groups at T1, T2 and T3

		T1(baseline)				T2(immediately after)				T3(endpoint)						Difference(I-C)	
		***N***	**Mean**	**SD**	***p*-value** ***t*-test**	***N***	**Mean**	**SD**	***p*-value** ***t*-test**	***N***	**Mean**	**SD**	**Mean difference (95%CI)**	***p*-value *t*-test**	**Cohen’s d**	**Mean difference (95%CI)**	*p*-value ANCOVA*
Total IDAS score	Intervention	69	80.23	22.67	0.351	62	87.60	29.24	0.570	60	97.98	27.15	12.94	0.006	0.50	14.43	0.000
	Control	83	83.82	24.27		74	85.01	23.62		65	85.05	24.48	(3.8, 22.1)			(7.34, 21.52)	
Scores for the 5 domains of the IDAS scale																	
Intervention characteristics	Intervention		20.10	6.77	0.209		22.50	7.55	0.681		25.07	7.07	2.70	0.030	0.39	3.59	0.001
	Control		21.54	7.20			22.00	6.60			22.37	6.67	(0.26, 5.13)			(1.55, 5.64)	
Outer setting	Intervention		9.64	3.65	0.120		11.21	4.26	0.639		12.25	3.98	1.51	0.025	0.41	2.27	0.000
	Control		10.58	3.72			10.91	3.29			10.74	3.48	(0.19, 2.83)			(1.08, 3.47)	
Inner setting	Intervention		23.07	7.91	0.543		25.44	9.33	0.456		28.60	8.07	4.31	0.003	0.55	4.21	0.001
	Control		23.82	7.19			24.34	7.78			24.29	7.54	(1.54, 7.07)			(1.67, 6.75)	
Characteristics of individuals	Intervention		10.83	2.85	0.887		11.31	4.30	0.672		12.62	3.92	1.89	0.007	0.49	1.77	0.002
	Control		10.90	3.86			11.03	3.15			10.72	3.76	(0.53, 3.26)			(0.64, 2.89)	
Process	Intervention		16.59	5.15	0.653		17.15	5.96	0.678		19.45	6.06	2.53	0.013	0.45	2.59	0.002
	Control		16.98	5.23			16.74	5.29			16.92	5.09	(0.55, 4.50)			(0.97, 4.20)	
		***N***	**Median**	**IQR**	***p*-value** **u-test**	***N***	**Median**	**IQR**	***p*-value** **u-test**	***N***	**Median**	**IQR**		***p*-value** **u-test**	**r**	**F**	*p*-value QANCOVA*
Level of understanding	Intervention	69	17	5.25-23	0.906	62	49	38-57.75	0.001	60	42	36-54		0.001	0.62	128.19	0.000
	Control	83	16	4.5-30.25		74	20	9-30		65	24	9-33.5					

*I-C* Intervention and control groups, *IQR* Interquartile range, *95CI* 95% confidence interval, *QANCOVA* Quede’s Nonparametric Analysis of covariance

^*^Covariate: baseline, affiliation and years of experience as a PHN

Furthermore, ANCOVA results adjusting for baseline scores, organization, and years of experience as a PHN showed significant differences favoring the intervention group across all measures. The adjusted mean difference in total IDAS scores between the intervention and control groups was 14.43 (95% CI 7.34–21.52, *p =* 0.000). Adjusted mean differences for the five domain scores of the IDAS were: intervention characteristics 3.59 (95% CI 1.55–5.64, *p =* 0.001), outer setting 2.27 (95% CI 1.08–3.47, *p =* 0.000), inner setting 4.21 (95% CI 1.67–6.75, *p =* 0.001), characteristics of individuals 1.77 (95% CI 0.64–2.89, *p =* 0.002), and process 2.59 (95% CI 0.97–4.20, *p =* 0.002).

The effect size of the total IDAS score was d = 0.50 for FAS and d = 0.48 for PPS. Among the five domains of the IDAS scale, the effect sizes were as follows: intervention characteristics (d = 0.39), outer setting (d = 0.41), inner setting (d = 0.55), characteristics of individuals (d = 0.49), and process (d = 0.45).

### Secondary outcome

The Table 3 also exhibits the results of the secondary outcome. The level of understanding, as analyzed by the U-test, was significantly higher in the intervention group compared to the control group at both T2 [median 49(IQR 38-57.75) vs. median 20(IQR 9‐30), *p =* 0.001] and T3 [median 42(IQR 36-54) vs. median 24(IQR 9-33.5), *p =* 0.001]. The endpoint or baseline difference was significantly higher in the intervention group compared to the control group (F = 128.19, *p =* 0.000). The effect size of the level of understanding was significant (r = 0.62).

### Process evaluation

Table 4 exhibits the duration of program viewing, ranging from 1 to 10 days. The most common length was three days (26.1%), followed by two days (20.3%), one day (14.5%), four days (14.5%), five days (7.2%), six days (7.2%), seven days (5.8%), and eight days (2.9%). Among the participants, 60.9% stopped viewing the program for one to three days. Only 1.4% of the participants viewed the program for 10 days.

**Table 4:** The length of program viewing

Day	N	(%)
1	10	(14.5)
2	14	(20.3)
3	18	(26.1)
4	10	(14.5)
5	5	(7.2)
6	5	(7.2)
7	4	(5.8)
8	2	(2.9)
10	1	(1.4)

*N=*69

The total scores for satisfaction with and confidence in the program are presented in Supplemental Table 1. The total satisfaction score was selected by 63 participants, ranging from 15 (full score) to 5 (dissatisfaction) points, with most scoring 15 (*n =* 20). The second highest number of respondents was 14 points (*n =* 12). There were 38 participants (60.3%) who selected between 13 and 15 points. Scores from 5 to 8 points were selected by 10 participants (15.9%). Scores from 9 to 12 were selected by 15 respondents (23.8%).

Total confidence scores were obtained from the same number of participants who selected 24 (full score) to 8 (lack of confidence) points. The highest number of participants selected 24 (*n =* 10). The second highest number of those who answered was 23 (*n =* 8) and 20 (*n =* 8) points. There were 39 participants (61.9%) who answered from 24 to 20 points, seven participants (11.1%) answered from 8 to 14 points, and seventeen participants (27%) answered from 15 to 19 points.

Comments were obtained from the participants after viewing each module (Table 5). Comments were categorized into groups of similar meanings and exhibited positive and negative reactions, together with any request about the program. The most common positive comment was about gaining basic competencies (*n =* 101). This was followed by willingness to grow (*n =* 51), satisfaction with program design (*n =* 45), and hope to utilize the program to learn how to find and use the evidence (*n =* 37). By contrast, 36 participants responded to requests to amend the program. The hope that coworkers and bosses would change their behaviors (*n =* 14), lack of confidence in implementing EBPH programs (*n =* 10), and difficulty in understanding (*n =* 8) were the most common comments.

**Table 5:** Positive comments and request or negative comments in the top4

Rank	Positive comments	N	Request or negative comments	N
1	Gaining Basic competencies	101	Request of amendment to the program	36
2	Growth willingness	51	The hope that co-workers and bosses change their behaviors	14
3	Satisfaction with program design	45	Lack of confidence in implementing EBPH programs	10
4	Hope to use this program to learn how to find or use the evidence	37	Difficult to understand	8

## Discussion

Our training program effectively enhanced the EBPH program implementation capacity of participants within four weeks. At T3, both the total IDAS score and scores across its five domains were significantly higher in the intervention group compared to the control group. When adjusted for participant demographics, the difference between endpoint and baseline scores was also notably larger in the intervention group.

The effect sizes for the total IDAS and its domains were generally moderate. Notably, the highest effect size was observed in the inner setting (d = 0.55). This domain focuses on organizational factors influencing program implementation, suggesting that our program effectively bolstered participants’ confidence in practical skills, yielding positive outcomes. Conversely, the lowest effect size was found in the intervention characteristics domain (d = 0.39), which assesses program suitability and adjustments for the target population [24]. Mastering skills in this area typically requires repeated exposure to deepen understanding, which may have been constrained by the program’s duration. Nevertheless, EPI-TRE demonstrated clear effectiveness in enhancing program implementation capacity among participants.

The level of understanding in the intervention group clearly exhibited a statistically significant increase at T2. Although the score declined slightly at T3, the training program achieved its objective. The study’s program provided the same multi-part content in different ways. Kelder et al. [32] argue that this progressive strategy facilitates learning new knowledge. In our study, this strategy appears to have had some impact on the total IDAS score.

Judging from the post-training comments, the lecture on how to find and utilize evidence appeared to have left a strong impression on the participants. Okamoto et al. found that only 11% of PHNs search for relevant academic articles [11]; however, participation in this program may have stimulated their interest in academic literature.

Kolb’s experimental learning model [30] elucidated that the effect of this program emerged four weeks post-intervention. Through the simulated experience of the EBPH program implementation, participants reflected on their current situation, developed a new concept of behavior at work, and applied this concept [30]. This may elucidate why the learning experience took effect after some time and not immediately. Consequently, the aim of the program was achieved. Our findings also support an existing review that reported that the knowledge and skills of participants generally enhance after attending an online training session [33].

A process evaluation of the training suggests that this program is appropriate for inexperienced PHNs, despite variations in the level of participation. The length of the program viewing was shorter than expected, but it did not prevent 60% of the participants from giving high scores regarding their levels of satisfaction and confidence in the program. Additional comments indicated a high level of satisfaction with the program. Comments indicating participants’ willingness to grow and proactive attitudes at work were also considered positive results of the program.

Simultaneously, however, numerous participants indicated their preference for fast-forwarding or printing programs to facilitate quick review, although the exclusion of these functions enabled us to evaluate the program’s effectiveness. Some participants also suggested the need for changes in the entire workplace environment. Successful program implementation requires the cooperation of everyone involved, including those in the workplace. Therefore, it is believed that all PHNs planning to implement an EBPH program should have access to this training. A prior study noted that capacity built at the individual level can be transferred to the entire group or organization concerned [34]. Furthermore, this training may have been too time-consuming or challenging for some participants to comprehend, as suggested by additional comments and the fact that several PHNs withdrew their participation after viewing the introduction to the program. We made systemic considerations to reduce the burden on trainees by timing the training to be held during the New Year holiday period when private time was available. Additionally, the training was designed as content that could be studied at the individual’s own pace and in small segments. However, owing to the re-emergence of new coronary infections, it appears that some aspects of the burden reduction did not work. No support system was introduced to verify the program’s effectiveness. Although most participants were satisfied with the content and system of the program, the researchers recognize the need to provide support to participants during web-based programs. However, in the case of capacity building, most training courses on D&I are offered at an advanced level, making it difficult for public health practitioners and policymakers to attend these courses. Minimal programs are reported to be available for beginners or practitioners [35, 36]. Therefore, we believe that this study’s program will be valuable for developing new PHNs.

Further training opportunities are necessary to support participants in implementing EBPH projects. In Japan, Yoshioka-Maeda et al. [20] developed an online program for mid-career PHNs, but their evaluation was limited to pre- and post-intervention measures and did not yield significant results. Programs aimed at researchers have highlighted the importance of mentorship for participants [37, 38]. In studies where behavioral changes were observed post-training (e.g., applying for funding or presenting at official forums), participants utilized networks established during the training or through online platforms, despite the training’s short duration [39]. An online community known as “Research to Reality (R2R)” serves as a hub for connecting researchers and communities [40]. It is necessary to provide continuous support for participants after the courses. Additionally, online connections between participants and researchers or among participants may compensate for the lack of mentors and increase the possibility of implementation.

### Limitations

This study encountered several limitations. First, the high attrition rate of 37% was possibly influenced by the significant waiting period between registration and the start of training, compounded by the onset of the eighth wave of the COVID-19 pandemic in Japan which coincided with the program’s launch. These factors may have contributed to reduced participant motivation and introduced inherent biases. Second, potential bias from participants sharing program details with third parties could not be ruled out, as adherence to confidentiality rules could not be verified. Finally, the program’s effectiveness was only evaluated over a four-week intervention period.

Moving forward, a subsequent step is to advocate for the adoption of this program among local government officials responsible for PHN training. Future research directions should include long-term program evaluations, the implementation of support systems to mitigate dropout rates, enhancements to the program structure such as medium-term implementation training, and the establishment of networking groups for PHNs interested in D&I.

## Conclusion

EPI-TRE, a web-based simulation program, effectively enhanced the implementation capacity of EBPH programs among inexperienced PHNs who reported positive impressions of the program. The simulations provided valuable opportunities for participants to gain practical experience in program implementation and reflect on their current practices in the workplace. This training program is expected to facilitate learning about program implementation for new PHNs.

## Supplementary Information

Supplementary Material 1.

## Data Availability

The datasets analyzed during the current study are not publicly available due to need further analysis but are available from the corresponding author on reasonable request. Access to materials for this program is available.

## Abbreviations

ANCOVA: Analysis of covariance
CFIR: Consolidated Framework for Implementation Research
D&I: Dissemination and implementation
EBPH: Evidence-based public health
EPI-TRE: Capacity Development Training Course for Evidence-based Program Implementation
FAS: Full analysis set
IDAS: Implementation Degree Assessment
PHNs: Public health nurses
PPS: Per protocol set
RED-Cap: Research Electronic Data Capture Sheet
