# Supplementary material for: Effect of web-based training on public health nurses’ program implementation capacity: a randomized controlled trial
Source: BMC Nurs. 2024 Sep 27;23:678. doi: 10.1186/s12912-024-02287-z (PMC11438300; doi:10.1186/s12912-024-02287-z)
Supplement: Supplementary file 1 — Supplementary Material 1. [file 12912_2024_2287_MOESM1_ESM.docx]

| Total scores for satisfaction | 5 | 6 | 7 | 8 | 9 | 10 | 11 | 12 | 13 | 14 | 15 |  |  |  |  |
| --- | --- | --- | --- | --- | --- | --- | --- | --- | --- | --- | --- | --- | --- | --- | --- |
| n (%) | 2(3.2) | 3(4.8) | 2(3.2) | 3(4.8) | 3(4.8) | 3(4.8) | 3(4.8) | 6(9.5) | 6(9.5) | 12(19.0) | 20(31.7) |  |  |  |  |
| Range:5-15 |  |  |  |  |  |  |  |  |  |  |  |  |  |  |  |
| Total scores for confidence | 8 | 9 | 10 | 13 | 14 | 15 | 16 | 17 | 18 | 19 | 20 | 21 | 22 | 23 | 24 |
| n (%) | 1(1.6) | 2(3.2) | 2(3.2) | 1(1.6) | 1(1.6) | 2(3.2) | 4(6.3) | 1(1.6) | 3(4.8) | 7(11.1) | 8(12.7) | 7(11.1) | 6(9.5) | 8(12.7) | 10(15.9) |
| Range:8-24 |  |  |  |  |  |  |  |  |  |  |  |  |  |  |  |
| 1 (Strongly disagree or disagree); 2 (Do not know); and 3 (Agree or Strongly agree) | | | | | | | | |  |  |  |  |  |  |  |

Supplement table1. The total scores for satisfaction and confidence (n=63)
